# Domain-specific cues improve robustness of deep learning-based segmentation of CT volumes

**DOI:** 10.1038/s41598-020-67544-y

**Published:** 2020-07-01

**Authors:** Marie Kloenne, Sebastian Niehaus, Leonie Lampe, Alberto Merola, Janis Reinelt, Ingo Roeder, Nico Scherf

**Affiliations:** 1 AICURA medical, Bessemerstrasse 22, 12103 Berlin, Germany; 2 Technische Fakultät, Universität Bielefeld, Universitätsstrasse 25, 33615 Bielefeld, Germany; 3 Institute for Medical Informatics and Biometry, Carl Gustav Carus Faculty of Medicine, Technische Universität Dresden, Fetscherstrasse 74, 01307 Dresden, Germany; 4 National Center of Tumor Diseases (NCT) Partner Site Dresden, Fetscherstrasse 74, 01307 Dresden, Germany; 5 Max Planck Institute for Human Cognitive and Brain Sciences, Stephanstrasse 1a, 04103 Leipzig, Germany

**Keywords:** Medical imaging, Three-dimensional imaging, Tomography, Image processing, Machine learning

## Abstract

Machine learning has considerably improved medical image analysis in the past years. Although data-driven approaches are intrinsically adaptive and thus, generic, they often do not perform the same way on data from different imaging modalities. In particular computed tomography (CT) data poses many challenges to medical image segmentation based on convolutional neural networks (CNNs), mostly due to the broad dynamic range of intensities and the varying number of recorded slices of CT volumes. In this paper, we address these issues with a framework that adds domain-specific data preprocessing and augmentation to state-of-the-art CNN architectures. Our major focus is to stabilise the prediction performance over samples as a mandatory requirement for use in automated and semi-automated workflows in the clinical environment. To validate the architecture-independent effects of our approach we compare a neural architecture based on dilated convolutions for parallel multi-scale processing (a modified Mixed-Scale Dense Network: MS-D Net) to traditional scaling operations (a modified U-Net). Finally, we show that an ensemble model combines the strengths across different individual methods. Our framework is simple to implement into existing deep learning pipelines for CT analysis. It performs well on a range of tasks such as liver and kidney segmentation, without significant differences in prediction performance on strongly differing volume sizes and varying slice thickness. Thus our framework is an essential step towards performing robust segmentation of unknown real-world samples.

## Introduction

The geometry of tumours, as described by, e.g. its size, shape or location, is a central clinical feature. Changes in these geometric characteristics are essential indicators of disease progression and can be used to measure treatment effects. An automated, quantitative assessment of these aspects and their changes from radiological images would yield an efficient and objective tool for radiologists to monitor the course of the disease. Thus, a reliable and accurate automated segmentation method is desirable to extract spatial tumour and organ characteristics from computed tomography (CT) volumes.

In recent years, convolutional neural networks (CNNs)^1^ became the state of the art method for image segmentation, as well as many other tasks in computer vision^2^, such as image classification, object detection and object tracking^3^. The applications of CNNs are diverse, but the general data handling or preprocessing is often very similar in each case since the feature extraction is performed internally by the CNN itself. Improvements in the application of CNNs for medical image processing often address changes in the neural network architecture, the training algorithm or the use case^4,5^. At the same time, most authors tend to ignore the data handling itself, treating medical images such as CT volumes the same way as grayscale or RGB images with additional dimensions.

However, this approach neglects prior information about the specific physical processes that underlie images acquisition and determine image contrast, possibly leading to suboptimal and sometimes inaccurate image analysis. For instance, most image formats map pixels on relative scales of a few hundred values. The voxels in CT volumes carry values from to the much wider Hounsfield scale^6^. This is a quantitative mapping of radiodensity calibrated such that the value for air is − 1,000  Hounsfield Units (HU) and for water 0 HU, with values in the human body reaching up to about 2000 HU (cortical bone). Therefore, in contrast to most standard images where pixel intensities themselves might not be meaningful, the actual grey values of CT volumes carry tissue-specific information^7^, and special consideration is required to leverage it.

The tissue-specific information also means, that CT data typically contains a range of values that are not necessarily relevant for a particular diagnostic question^8,9^. Thus, when radiologists inspect CT volumes for diagnosis, they typically rely on windowing, i.e. they restrict the range of displayed grey values to focus the image information to relevant values. CNN-based image segmentation frameworks rarely include such potentially essential steps from the expert workflow, assuming that the data only has to be normalised and the network will then learn by itself to focus on the relevant image regions.

In this paper, we address the challenges of a clinically meaningful CT volume processing and present a domain-specific framework for CNN based image segmentation. The proposed framework is inspired by insights into the data acquisition and the diagnostic process performed by the radiologist, addressing, in particular, the spatial information in CT volumes and the use of the HU scale.

From a technical point of view, our primary aim is to obtain a reliable segmentation result (i.e. a low variation in segmentation quality across a variety of inputs) rather than pursuing high accuracy only. Robustness is an essential requirement when we want to use a segmentation model as part of a (semi-)automated analysis process. In this case, significant segmentation errors can go undetected as we might not directly analyse the actual segmentation result but only downstream results derived from it. Thus, we require algorithms to analyse each volume consistently and without significant differences in output quality. To estimate the robustness of the model output, we evaluate the standard deviation of the Dice score.

We evaluated the framework with different neural architectures: We implemented a mixed-scale dense convolutional neural network (MS-D Net)^10^ with dilated convolutions and the nnU-Net^11^ (a modified U-Net)^12^ with traditional scaling operations. We consider both a 2D-CNN and a 3D-CNN implementation for each architecture. Finally, we show that an ensemble CNN allows combining the volumetric information leveraged in 3D-CNNs with the proportionally higher value of each segmented voxel in the 2D-CNNs training process, resulting in more accurate results.

The typical assumption behind cross-validation is that the data set is representative of yet to be seen real data, and the test or validation sample should also reflect this. Thus, we would usually balance all folds, so they contain typical samples and also possible outliers. However, we want to assess how robust the trained models are, and thus here we do not randomly mix the folds. Instead, we assign each sample to a fold depending on the number and thickness of its slices. This way, we will always have samples in the test set that are independent of the training data, and we simulate the worst-case scenario for the application in the clinical environment. In order to make the results reproducible, we use open datasets for training and evaluation. We train and validate the CNN-models for kidney tumour segmentation on the dataset of the 2019 Kidney Tumor Segmentation Challenge^13^. For the liver segmentation, we use the dataset of the CHAOS—Combined (CT-MR) Healthy Abdominal Organ Segmentation Challenge^14^.

It seems like the rise of deep learning methods in medical image analysis has split the community into two factions: those who embrace such methods and those who do not trust them. We think that to apply deep learning in a clinical setting, the CNN architectures and the entire workflow for data processing and augmentation need to be adapted, requiring considerable knowledge of the diagnostic question and the imaging modality at hand. In this work, we want to show that in order to build clinically applicable CNN-based frameworks, we require different expertise and input from technical and medical domain experts.

**Fig. 1:**
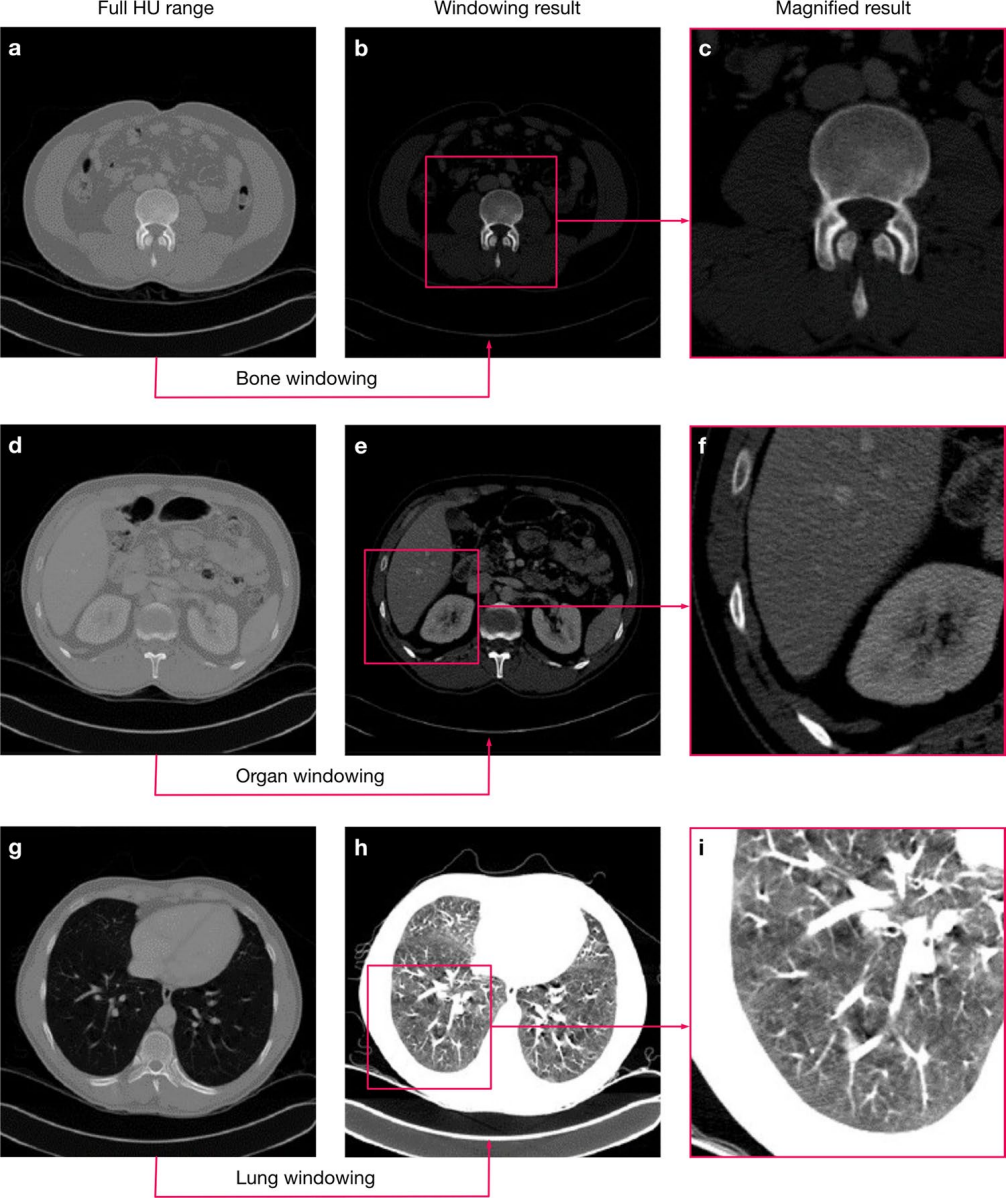
Windowing highlights tissues of interests and reduces the complexity of background structures. Three examples for the use of case-oriented windowing for bones (**a**–**c**), organs (**d**–**f**), and lungs (**g**–**i**). We used the organ oriented windowing in this work, while we show the other two examples for comparison. We derived the intensity windows for CNN processing by slightly extending the standard ranges used by radiologists in practice to allow for uncertainties in the exact ranges.

## Methods

In the following, we describe the data processing and augmentation in “Preprocessing and augmentation” section, and the network architectures in “Architecture” section. The preprocessing includes volume shape reduction and grey-value windowing. The proposed augmentation addresses the scarcity of data, to provide additional samples for the training procedure. For the CNN architectures, we consider two models to compare dilated convolutions (MS-D) to traditional scaling operations (U-Net). We further explain the construction of the stacked CNN model. The training procedure for the two considered architectures is described in “Training” section.

### Preprocessing and augmentation

In order to ensure adequate data quality in the training process for each model, we adapt the data preprocessing and augmentation for CT data. We developed the preprocessing based on the dataset of the KiTS Kidney Tumor Segmentation Challenge^13^ and the dataset of the CHAOS—Combined (CT-MR) Healthy Abdominal Organ Segmentation Challenge^14^. However, the same can be applied to any other CT dataset with minor changes.

#### Image preprocessing

We adapted the image normalisation from^11^ to better suit real-world applications. To reduce the complexity and optimise the dynamic range, we apply a windowing to each volume by clipping the voxels grey value range to a (0.6, 0.99) percentile range that corresponds to the window a radiologist would use for decision-making. For other segmentation problems, the percentiles must be adjusted to fit the intensity distribution of the relevant body parts (We show examples in Fig. 1). We then normalise the windowed data using the z-score using the intensity statistics (mean, standard deviation) from a random subset of the data set. Using the intensity statistics from the entire dataset might give slightly better normalisation results, but this approach would not always reflect the typical conditions in a clinical environment. Often, we continuously collected the image data over time and thus, only a subset of the data is available before the training process starts.

**Fig. 2:**
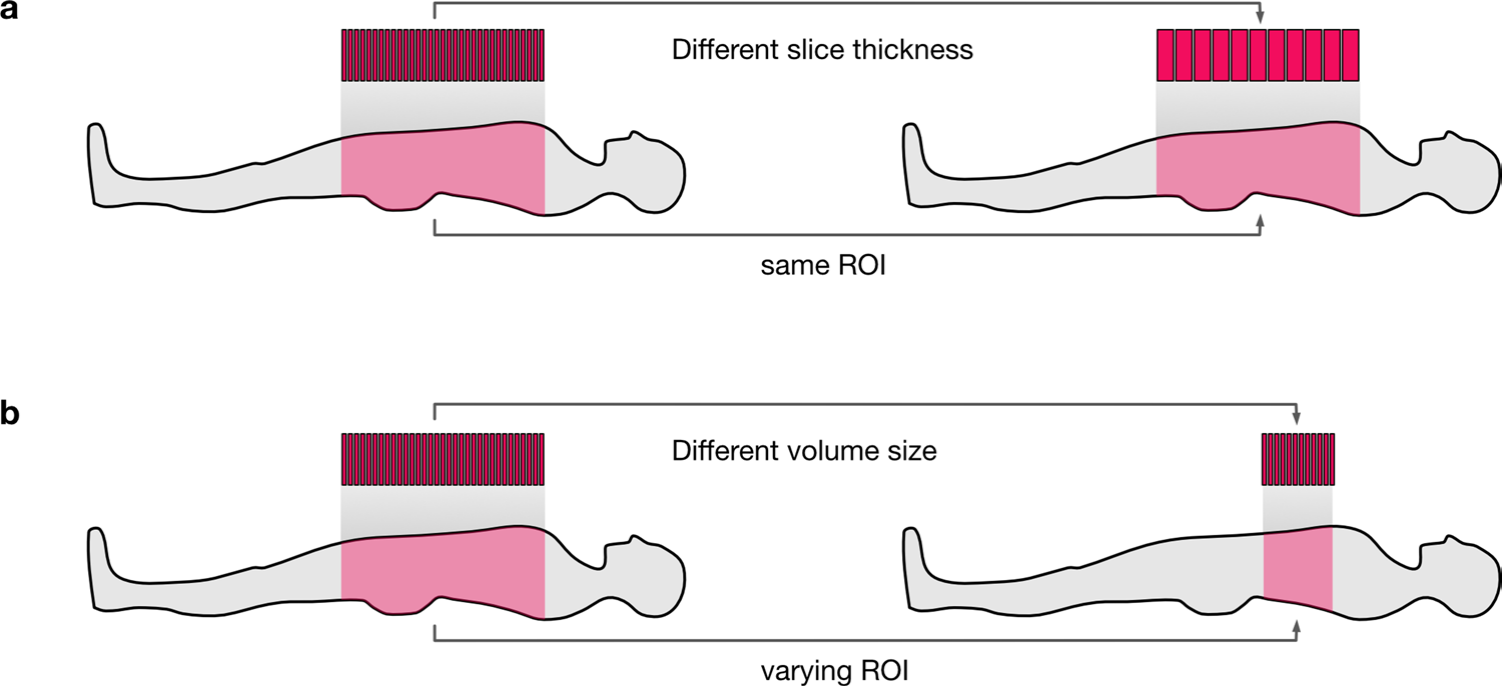
Differences in CT scanning configurations pose challenges for CNN-based segmentation. (**a**) Varying slice thickness maps the same anatomical region of interest to different numbers of slices. Thicker slices reduce the scan time for larger regions of interest, but 3D details and semantic context can be lost. (**b**) Volume size varies depending on the chosen region of interest. Normalising to a standardised volume size then requires strong interpolation.

In order to save costs and time and reduce exposure to radiation in CT, the radiologist typically confines an acquisition to the region of interest (ROI) (Fig. 2). This ROI is typically defined liberally not to miss an area that is potentially relevant to the diagnosis. Thus, in a clinical setting, the number of acquired slices in a CT volume varies considerably. The varying slice number poses a challenge to the application of standard CNN pipelines which often assume a regular data sampling. To standardise the data, we decided to reduce each volume to 16 slices as we do not need to upsample volumes that contain only a few slices. Instead, our method selects slices at random positions from each volume, and by repeating the sampling process per volume, we also get a simultaneous data augmentation effect. We exclude background slices during the training phase since these are also not considered in the test phase. We observed that increasing the number of slices did not yield better results, which is consistent with the observation that most CNNs only use a small semantic context for decision making^15,16^.

In order to save GPU memory, we downsampled each slice from $512 \times 512$ voxels to $128 \times 128$ voxels as in our experiments larger slice sizes did not yield better segmentation performance.

#### Image augmentation

As additional augmentation steps we used image noising with a normally distributed noise map, a cluster-wise voxel intensity range shift, slice skipping and slice interpolation to address potential variation in the CT acquisition process (Fig. 2). We introduce a cluster-wise voxel intensity range shift (CWVRS) to make the network more robust to slight, vendor—or patient-specific variations in the exact tissue intensities that lead to different spatial patterns after windowing. To simulate these changes, we slightly increase, decrease, or shift the exact intensity windows and rescale the intensity values in this range during augmentation. The base intensity windows were defined in advance and correspond to the typical intensity ranges used in routine radiological workflows (Supplementary Table S1 online). We further rotated the images by a random angle (maximum of 16°) to simulate the inevitable variability in patient positioning, that occurs in clinical routine despite fixation. These augmentation steps should more realistically model the expected data variation when applying the deep learning models in clinical practice.
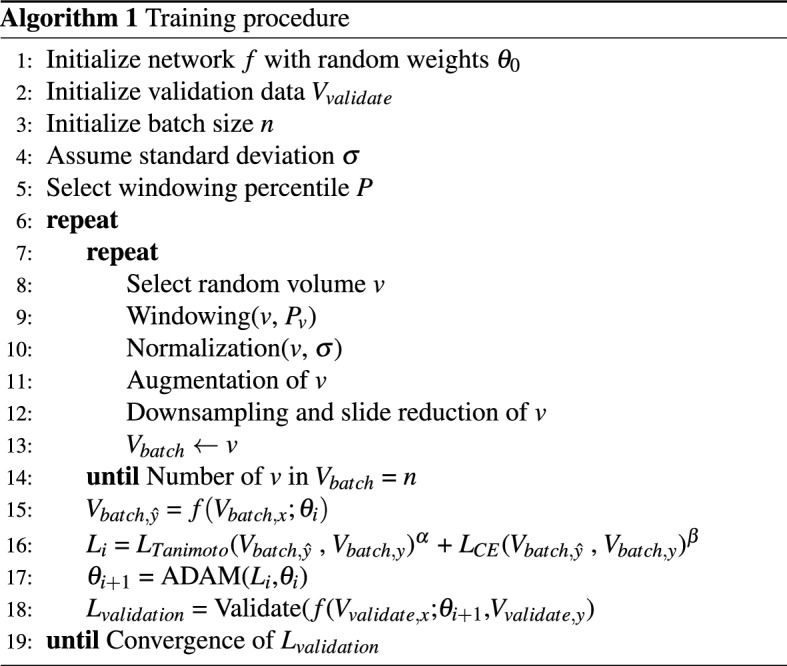


### Architecture

To demonstrate the independence of our preprocessing and augmentation framework from the concrete underlying neural architecture, we compared two conceptually different CNN models. The first architecture we consider here is a modified version of the widely-used U-Net called nnU-Net^11^. This architecture extends the original U-Net architecture^12^ by replacing batch normalization^17^ with instance normalization^18^ and ReLUs with LeakyReLU units of slope 1e$-2$^19^. For comparison, we chose the mixed-scale dense convolutional neural network (MS-D net)^10^. We modified it in the same way as the U-Net to remove the influence of the activation function in our comparison. We have chosen these two rather extreme variants of CNNs to compare the traditional down- and upscaling flow with the parallel multi-scale approach using dilated convolutions.

In clinical diagnoses, the radiologist locates the tumour and relevant adjacent structures not only by examining the individual slice but also the adjacent slices. Thus, a 3D CNN might seem like the obvious choice in order to not lose the spatial information from the 3D context. However, previous work has clearly shown that 3D segmentation methods perform worse than 2D approach when the data is anisotropic^20,21^, which is regularly the case in medical imaging. Another reason why medical image segmentation with 3D CNNs often proves challenging is the variable number of slices per volume. The slice number depends on various external factors like body region under investigation, diagnostic question, different size of the subjects and other trade-offs between data quality, minimal scanning time and radiation exposure. Thus somewhat counterintuitively, 3D CNNs do not necessarily perform better than 2D versions in many circumstances, and robust models should consider both options.

Finally, we combined different models into a single, stacked CNN model to leverage the different strengths of each architecture as ensemble methods showed superior performance in several detection tasks^22–24^. For the kidney-tumour segmentation, we stacked a set of 3D MS-D Nets trained to classify voxels into kidney and background (without a distinction between the healthy kidney tissue and the tumour tissue), and a set of 2D nnU-Nets trained to perform classification into healthy tissue, tumour and background. For the liver segmentation, both models perform binary classification of voxels into liver and background.

### Training

We trained all networks independently from scratch. The overall training procedure shown in Algorithm 1 was implemented in Python with Tensorflow 1.14 and performed on an IBM Power System Accelerated Compute Server (AC922) with two NVIDIA Tesla V100 GPUs. This setup allowed us to parallelise the experiments, but our proposed approach also works on typical systems with an NVIDIA GTX 1080.

In each epoch, the volumes of a randomly selected batch are preprocessed and augmented (lines 9–12). We used a batch size of 28 for the 2D networks, while we had to reduce the batch size to 1 (stochastic gradient descent) for the 3D versions of the modified architectures. We use data augmentation in 80% of the training batches for 3D and 90% of training batches in 2D. We applied the intensity range shift to 20% of data in both cases.

To update the weights $\theta _{i}$ of the neural network function *f*, we used the ADAM optimisation with the parameter configuration proposed in^25^. Our loss function *L* (line 16 in Algorithm 1) is a combination of the Tanimoto loss $L_{Tanimoto}$ and the categorical crossentropy $L_{CE}$, weighted by $\alpha = 0.6$ and $\beta = 0.4$ respectively. We implemented the Tanimoto loss as shown in Eq. 1, where $\hat{y}\in \hat{Y}$ denotes the set of predicted voxel-wise annotations and $y\in Y$ denotes the set of ground truth voxel-wise annotations. The advantage of the Tanimoto coefficient is that it treats each class independently and is thus particularly suitable for problems with a high class imbalance which is typically the case in medical imaging. However, this also leads to a maximum error if a particular class does not occur in the current sample. This effect is attenuated by the smooth factor *smooth*. We empirically chose a small *smooth* of 1e−5. A more detailed discussion is given in^26^.

1 $$\begin{aligned} L_{Tanimoto}(\hat{Y}, Y) = 1 - \frac{\hat{Y}Y + smooth}{\vert \hat{Y}\vert ^2+\vert {Y}\vert ^2 - \hat{Y}Y + smooth} \end{aligned}$$

## Evaluation

**Fig. 3:**
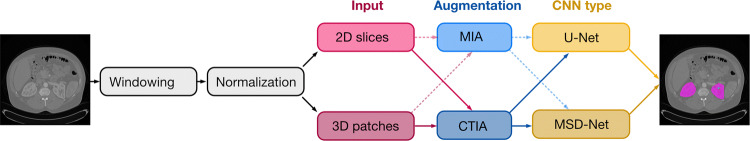
Overview of the different segmentation workflows that we considered in our experiments. The arrows (both solid and dashed) indicate different combinations of input dimension, augmentation and CNN architectures. The solid arrows specifically highlight the best combination.

We compared our domain-specific augmentation to the state-of-the-art multidimensional image augmentation method from^27^ implemented in TensorFlow across different image dimensionalities and neural architectures (Table 1). We illustrate the overview of the different experiments in Fig. 3.

**Table 1:** Comparison of the CT-specific image augmentation (CTIA) and the multidimensional image augmentation (MIA).

Transformation	CTIA	MIA
Spatial transformation	Random patch extraction	Scaling
	Slice skipping	Random rotation
	Slice interpolation	Without restriction
	Random rotation (maximum of 16°)	Image shearing
		Cropping
Intensity transformations	Cluster-wise voxel intensity range shift	Gamma-corrections
		Contrast
	Image noising with Gaussian noise	Brightness
		Image noising
		with Gaussian noise

We kept the same normalisation and windowing preprocessing steps in both cases. Since the normalisation and windowing of the CT volume reduce the anatomical structures that are visible in the volume, some parts of (e.g. the skull) with values outside the range become background after windowing. This effect leads to different results in cropping and selection of slices.

An exact comparison of the two preprocessing approaches would not be possible under these circumstances, because we would effectively compare pipelines that work on images of different sizes. To show that preprocessing does not negatively influence results, we show an experiment without windowing in Supplementary Table S2 online and Supplementary Table S3 online. However, we cropped the images to the same regions as obtained from windowing to ensure comparability and to avoid any performance benefits related to smaller image crops. We point out that using windowing as a preprocessing step considerably reduced training time (number of epochs until convergence) in our experiments. This effect is likely due to the suppression of anatomical regions that are not of interest, and thus windowing effectively decreases the complexity of the background regions that the network has to learn.

For comparing the effect of image augmentation across different segmentation models, we implemented both CNN architectures in a 2D and 3D version and evaluated each model in 5-fold cross-validation. To include the influence of edge cases in our validation, we sorted the data according to the number of slices, so we could always validate the models on CT volumes that did not occur in the training data set in a similar form. We numerically evaluated the model predictions volume-wise using the Dice score, as shown in Eq. 2 using the same annotation as in Eq. 1. We report the resulting scores averaged over volumes and cross-validation folds for the kidney tumour segmentation in Table 2 and for the liver segmentation in Table 3.

2 $$\begin{aligned} s_{Dice}(\hat{Y}, Y) = \frac{2\hat{Y}Y}{\vert \hat{Y}\vert ^2+\vert {Y}\vert ^2} \end{aligned}$$

The results show that the average prediction performance of models trained with CT-specific image augmentation is on par with the performance of models using multidimensional augmentation. However, the CT-specific preprocessing yields stable results whose standard deviation is an order of magnitude lower than the state-of-the-art multidimensional approach from^27^. Supplementary Fig. S2 online and Supplementary Fig. S3 online highlight the clinical relevance of these more stable predictions by showing the impact of a reduced standard deviation in numerical segmentation accuracy on actual segmentation results. Our results further confirm existing empirical findings that including 3D spatial information in models does not necessarily lead to a better segmentation performance for anisotropic data.

**Fig. 4:**
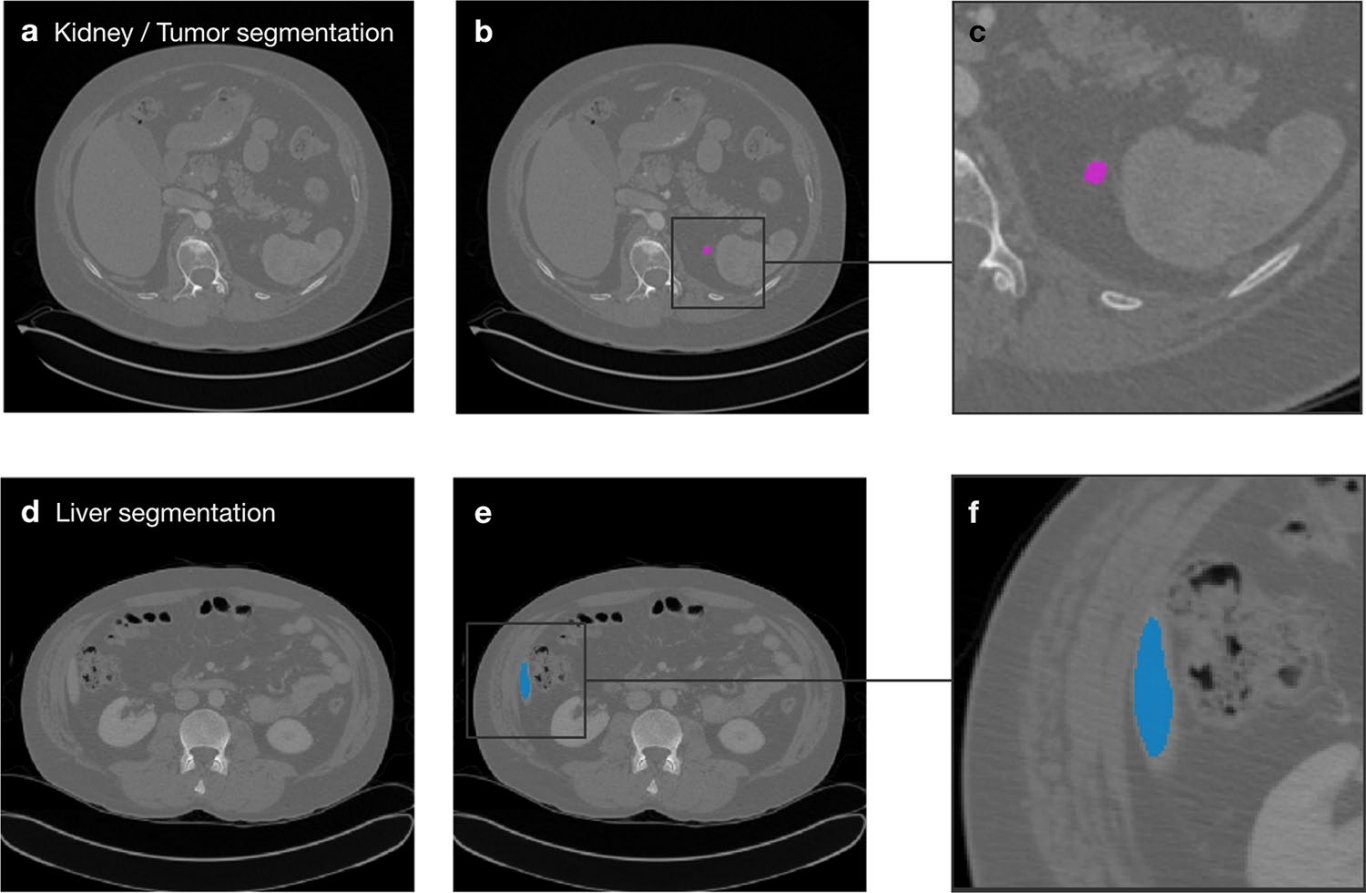
Examples of challenging 2D segmentation cases. Examples are shown for kidney and tumor segmentation (**a**) and liver segmentation (**b**). Segmentation errors typically occur more frequently in the first and last slice of the ROI.

We found that performance varied across the different neural architectures. For the kidney segmentation, our findings indicate that multi-scale architecture could detect whole objects very well as the 3D MS-D Net shows fewer background errors in binary segmentation. However, the finer distinction between foreground classes (kidney and tumour tissue) worked comparatively poorly as. For liver segmentation, we found that the MS-D Net generally led to more segmentation errors. However, the MS-D Net errors typically differ from the type of segmentation errors of the U-Net approach. In particular, slices with only small regions of interest (shown in Fig. 4) pose a challenge.

Since the errors of the MS-D Net are complementary to the errors of the nnU-Net for both cases, a stacked CNN leads to consistently better results, as it can learn to balance the strengths and weaknesses of the different models. Here, we constructed a stacked CNN consisting of a set of 3D MS-D Nets and a set of 2D nnU-Nets trained with CT-specific image augmentation. For each set, we selected the top-5 models based on their validation score in the previous experiment. The stacked ensemble of neural network predictor consistently delivered the most accurate and stable predictions by combining the different individual strengths of their members (see Tables 2, 3).

**Table 2:** Results for the kidney tumor segmentation: Total Dice scores are reported (mean ± stdv.) for each segmentation class, the different architectures and input dimensionalities (2D and 3D). Each approach is validated with the multidimensional image augmentation (MIA) for Tensorflow and with our CT-specific image augmentation (CTIA).

		Kidney	Tumor	Total
nnU-Net + MIA	2D	$\; 0.962 \pm 0.006$	$\; 0.840 \pm 0.013$	$\; 0.929 \pm 0.009$
nnU-Net + CTIA	2D	$\; 0.961 \pm 0.001$	$\; 0.844 \pm 0.007$	$\; 0.931 \pm 0.002$
nnU-Net + MIA	3D	$\; 0.960 \pm 0.012$	$\; 0.839 \pm 0.021$	$\; 0.929 \pm 0.014$
nnU-Net + CTIA	3D	$\; 0.960 \pm 0.002$	$\; 0.841 \pm 0.008$	$\; 0.925 \pm 0.003$
MS-D Net + MIA	2D	$\; 0.950 \pm 0.011$	$\; 0.774 \pm 0.022$	$\; 0.913 \pm 0.014$
MS-D Net + CTIA	2D	$\; 0.950 \pm 0.001$	$\; 0.779 \pm 0.009$	$\; 0.914 \pm 0.003$
MS-D Net + MIA	3D	$\; 0.947 \pm 0.012$	$\; 0.764 \pm 0.024$	$\; 0.906 \pm 0.018$
MS-D Net + CTIA	3D	$\; 0.948 \pm 0.002$	$\; 0.765 \pm 0.009$	$\; 0.907 \pm 0.003$
Stacked CNN + MIA		$\; 0.968 \pm 0.008$	$\; 0.841 \pm 0.011$	$\; 0.943 \pm 0.008$
Stacked CNN + CTIA		$\; 0.968 \pm 0.001$	$\; 0.845 \pm 0.004$	$\; 0.947 \pm 0.002$

**Table 3:** Results for liver segmentation: Total Dice score (mean ± stdv.) for the different architectures and input dimensionalities (2D and 3D). We validated each approach with the multidimensional image augmentation (MIA) for Tensorflow and with our CT-specific image augmentation (CTIA).

		Total
nnU-Net + MIA	2D	$\; 0.974 \pm 0.031$
nnU-Net + CTIA	2D	$\; 0.978 \pm 0.001$
nnU-Net + MIA	3D	$\; 0.941 \pm 0.027$
nnU-Net + CTIA	3D	$\; 0.944 \pm 0.014$
MS-D Net + MIA	2D	$\; 0.961 \pm 0.032$
MS-D Net + CTIA	2D	$\; 0.964 \pm 0.002$
MS-D Net + MIA	3D	$\; 0.942 \pm 0.037$
MS-D Net + CTIA	3D	$\; 0.942 \pm 0.004$
Stacked CNN + MIA		$\; 0.976 \pm 0.021$
Stacked CNN + CTIA		$\; 0.980 \pm 0.001$

## Conclusion

**Fig. 5:**
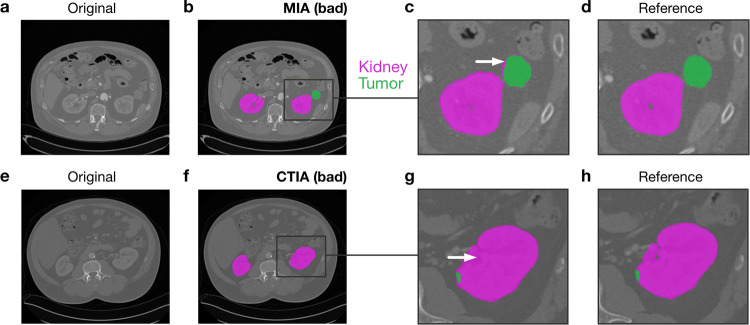
The influence of data augmentation on segmentation quality. Typical examples of low-quality segmentation results of a 2D U-Net trained with (**a**–**d**) MIA and (**e**–**h**) CTIA. The arrows in the magnified results in (**c**) highlight incorrect tissue classifications obtained by training with MIA. The reference segmentation is depicted in (**d**) for comparison. In contrast, segmentation errors in the pipeline trained with CTIA are typically limited to the size of the segmented region (**g**) as compared to the reference shown in (**h**). Both examples demonstrate cases with an accuracy of less than one standard deviations below the mean for the respective pipeline. The examples were selected randomly from a pool of examples of the same quality. The effects shown occur for 2D and 3D segmentation models alike (see Supplementary Fig. S1 online) for further examples.

In this work, we propose a robust machine learning framework for medical image segmentation addressing the specific demands of CT images for clinical applications. Our analysis focused on the often neglected influence of preprocessing and data augmentation on segmentation accuracy and stability. We systematically evaluated this framework for two different state-of-the-art CNN architectures and 2D and 3D input data, respectively. In line with previous findings^20,21^, our results show that 3D spatial information does not necessarily lead to better segmentation performance in particular concerning detailed, small-scale image structures. In our experiments, the types of segmentation errors varied between neural network models, and we showed that a stacked CNN model combining a top-*n* selection from each model indeed outperformed all other approaches considered in this work. Thus, our findings suggest an ensemble approach as an effective way to achieve more robust and thus, reliable performance in a routine setting. Most importantly, our work shows that a domain-specific data augmentation scheme can yield highly robust segmentation results with an order of magnitude lower variation while maintaining the same average segmentation accuracy as the general-purpose state-of-the-art approach. The improvements are independent of the underlying CNN architecture. Although the reduced variability in the Dice score might seem like a minor numerical effect, the individual differences in segmentation quality do have a clinical impact. Errors of models trained with CT-specific image augmentation are mostly limited to minor differences in the size of segmented regions and finer details in the outlines. Models trained with multidimensional image augmentation show more severe errors like unrecognised tumour parts or misclassified tissue regions (Fig. 5). In particular small tumours or tumour regions that appear disconnected from to the kidney in the image are typically not recognised correctly. These cases occur even for 3D segmentation models in particular when the distance between the slices becomes higher, and the network does not properly learn the 3D connectivity of the tissue. Thus, the slice interpolation and slice skipping in the data augmentation have a substantial impact and lead to more reliable results. Furthermore, we found the cluster-wise voxel intensity range shift to improve segmentation stability. We speculate that this augmentation step might help focus the feature extraction in the networks more on reliable spatial structures in the image and less on actual voxel intensities.

Robust and simple machine learning pipelines such as the one outlined in this paper have the potential to improve clinical nephometry substantially. Existing scores used in clinical routine have a poor predictive power^13^ and massively reduce the underlying information contained in CT volumes. The improved characterisation of kidney tumours through a more efficient, objective and reliable segmentation, should yield better clinical evaluation, better prediction of clinical outcomes, and ultimately a better treatment of the underlying pathology. In our view, to pave the way to routine clinical applications of machine learning methods for diagnostic decision support, we must focus on improving the robustness and reliability of our segmentation methods. We advocate to increase the interpretability and acceptance of those models by explicitly incorporating prior knowledge, for example by recapitulating processing steps from clinical workflows. As a step into this direction, our work addresses fundamental methodological challenges in automated segmentation of CT volumes for medical use, to yield reliable organ and tumour segmentation.

## Supplementary information

Supplementary information

## Data Availability

A preprint version of this article is available at: https://arxiv.org/abs/1907.10132.
