# Supplementary material for: Domain-specific cues improve robustness of deep learning-based segmentation of CT volumes
Source: Sci Rep. 2020 Jul 1;10:10712. doi: 10.1038/s41598-020-67544-y (PMC7329868; doi:10.1038/s41598-020-67544-y)
Supplement: Supplementary file 1 — Supplementary information [file 41598_2020_67544_MOESM1_ESM.pdf]

Domain-specific cues improve robustness of deep learning-based segmentation of CT volumes.

Marie Kloenne, Sebastian Niehaus, Leonie Lampe, Alberto Merola, Janis Reinelt, Ingo Roeder, and Nico Scherf

Supplementary Information

Supplementary Table S 1: The voxel clusters for the cluster-wise voxel range shift.

Organ	Width	Level
mediastinum	360	50
soft tissues (abdomen)	400	50
liver	150	30
soft tissues (spine)	250	50

Supplementary Table S 2: Results for the kidney tumor segmentation without windowing: Total Dice scores are reported (mean ± stdv.) for each segmentation class, the different architectures and input dimensionalities (2D and 3D). Each approach is validated with the multidimensional image augmentation (MIA) for Tensorflow and with our CT-specific image augmentation (CTIA). To avoid predicting on differently sized slices and to ensure that the results be compared, cropping is applied in the same way as in the experiments with windowing.

		Kidney	Tumor	Total
nnU-Net + MIA	2D	0.961 ± 0.005	0.840 ± 0.015	0.928 ± 0.010
nnU-Net + CTIA	2D	0.961 ± 0.002	0.843 ± 0.006	0.930 ± 0.002
nnU-Net + MIA	3D	0.959 ± 0.011	0.839 ± 0.019	0.928 ± 0.014
nnU-Net + CTIA	3D	0.961 ± 0.002	0.840 ± 0.007	0.925 ± 0.004
MS-D Net + MIA	2D	0.949 ± 0.012	0.773 ± 0.024	0.912 ± 0.016
MS-D Net + CTIA	2D	0.949 ± 0.001	0.774 ± 0.009	0.913 ± 0.004
MS-D Net + MIA	3D	0.945 ± 0.014	0.762 ± 0.024	0.904 ± 0.019
MS-D Net + CTIA	3D	0.947 ± 0.002	0.765 ± 0.009	0.906 ± 0.003
Stacked CNN + MIA		0.967 ± 0.009	0.842 ± 0.010	0.943 ± 0.009
Stacked CNN + CTIA		0.967 ± 0.001	0.846 ± 0.005	0.947 ± 0.001

Supplementary Table S 3: Results for liver segmentation without windowing: Total Dice score (mean ± stdv.) for the different architectures and input dimensionalities (2D and 3D). Each approach is validated with the multidimensional image augmentation (MIA) for Tensorflow and with our CT-specific image augmentation (CTIA). To avoid predicting on differently sized slices and to ensure that the results be compared, cropping is applied in the same way as in the experiments with windowing.

		Total
nnU-Net + MIA	2D	0.968 ± 0.028
nnU-Net + CTIA	2D	0.969 ± 0.001
nnU-Net + MIA	3D	0.929 ± 0.029
nnU-Net + CTIA	3D	0.932 ± 0.013
MS-D Net + MIA	2D	0.959 ± 0.033
MS-D Net + CTIA	2D	0.963 ± 0.002
MS-D Net + MIA	3D	0.936 ± 0.039
MS-D Net + CTIA	3D	0.937 ± 0.004
Stacked CNN + MIA		0.975 ± 0.020
Stacked CNN + CTIA		0.978 ± 0.001

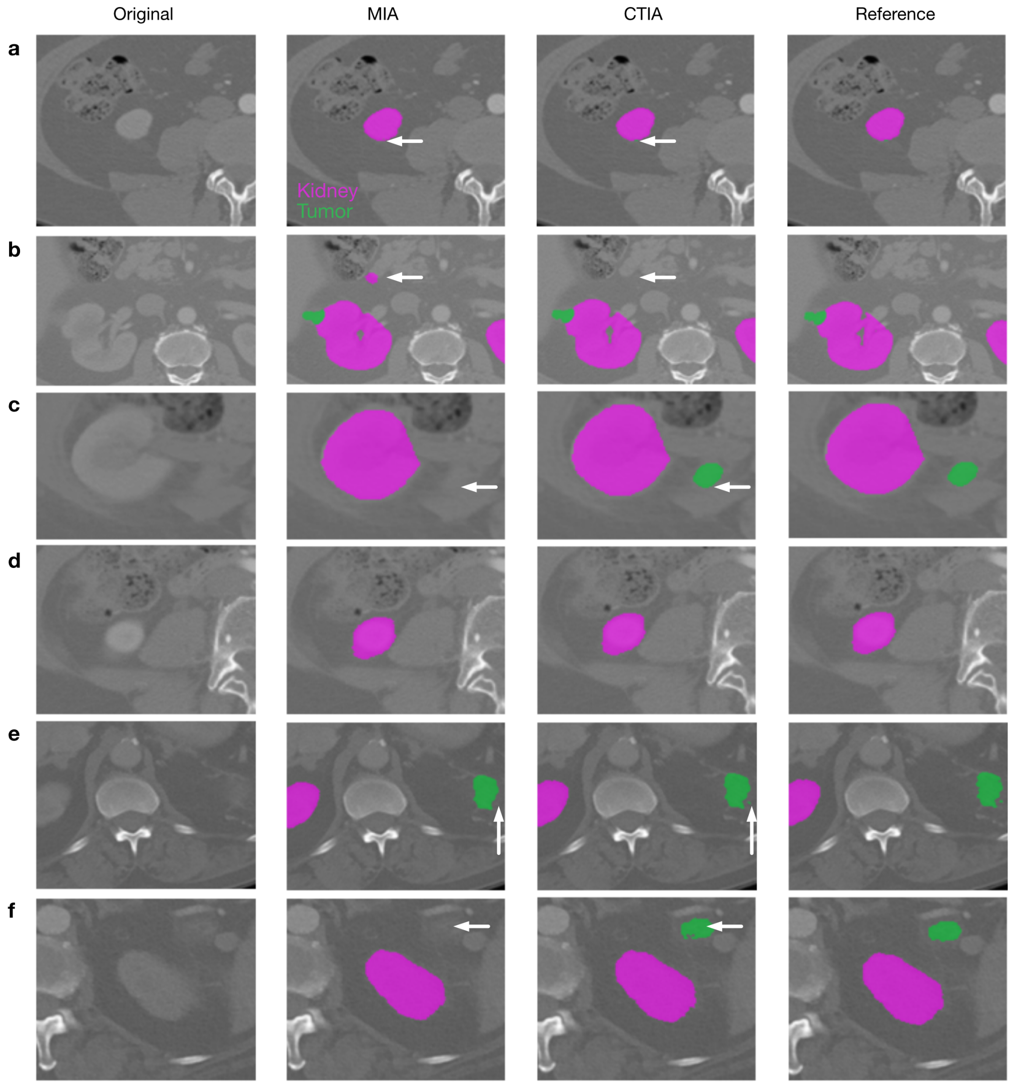

Supplementary Figure S 1: Randomly selected bad examples of segmentations of 2D U-Nets trained with MIA. This selection indicates that the strong segmentation errors are usually misclassifications. The examples **a**,**c** **f** show undetected kidney tumours and the example **e** shows an instance in which a severed tumor is not recognized. Another misclassification is the classification of tissue in the background as kidney **b**. Example **d** demonstrates only slight errors in the size of the volume, but these are more significant for the dice score, since only one class exists in the ground truth.

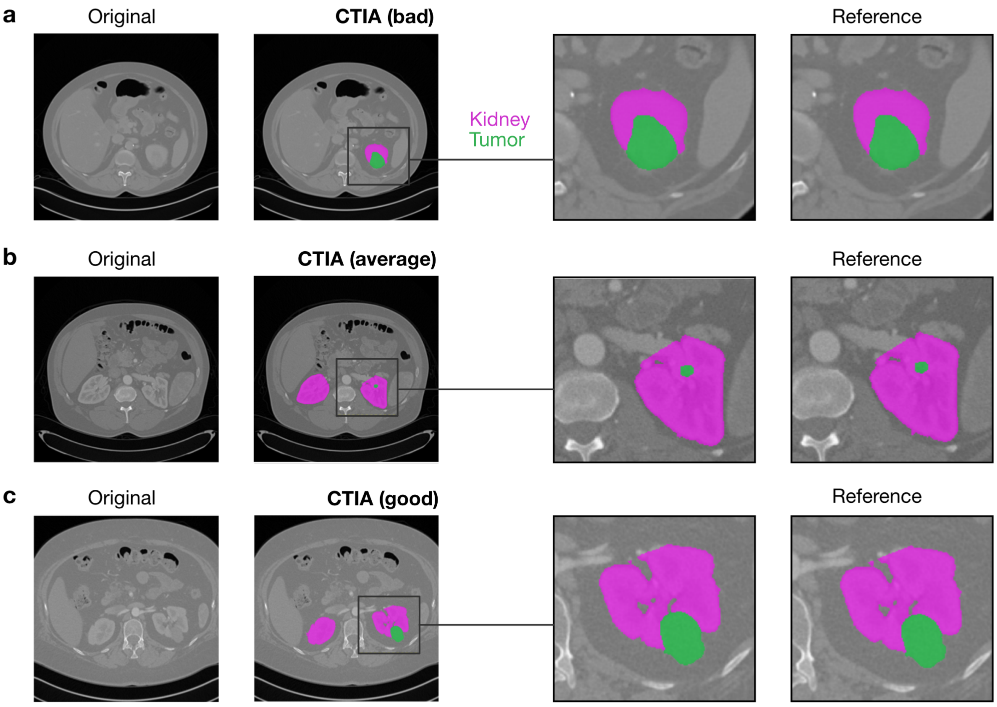

Supplementary Figure S 2: Examples of the segmentation quality of a 2D U-net trained with CTIA. The examples have the following dice scores: **a**: $s_{Dice,Bad} \approx 0.920$, **b** : $s_{Dice,Average} \approx 0.929$, **c** : $s_{Dice,Good} \approx 0.938$.

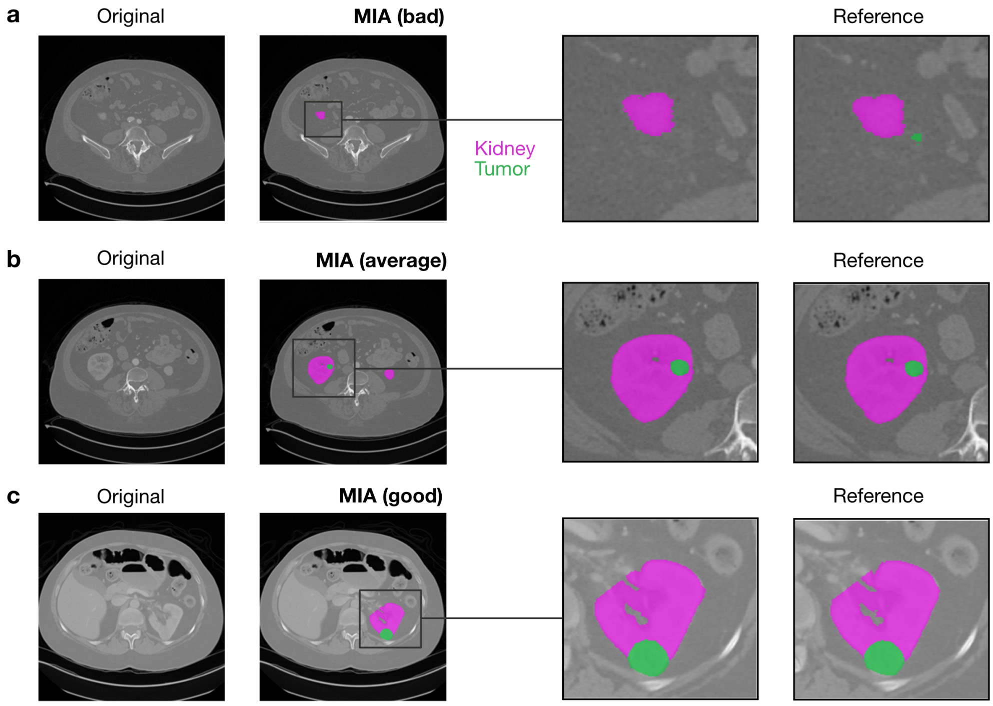

Supplementary Figure S 3: Examples of the segmentation quality of a 2D U-net trained with MIA. The examples have the following dice scores: **a**: $s_{Dice,Bad} \approx 0.929$, **b** : $s_{Dice,Average} \approx 0.931$, **c** : $s_{Dice,Good} \approx 0.933$.
